# Experimental quantum-enhanced kernel-based machine learning on a photonic processor

**DOI:** 10.1038/s41566-025-01682-5

**Published:** 2025-06-02

**Authors:** Zhenghao Yin, Iris Agresti, Giovanni de Felice, Douglas Brown, Alexis Toumi, Ciro Pentangelo, Simone Piacentini, Andrea Crespi, Francesco Ceccarelli, Roberto Osellame, Bob Coecke, Philip Walther

**Affiliations:** 1 University of Vienna, Faculty of Physics, Vienna Center for Quantum Science and Technology (VCQ), Vienna, Austria; 2 University of Vienna, Faculty of Physics, Vienna Doctoral School in Physics (VDSP), Vienna, Austria; 3 Quantinuum, Oxford, UK; 4 Dipartimento di Fisica, Politecnico di Milano, Milan, Italy; 5 Istituto di Fotonica e Nanotecnologie, Consiglio Nazionale delle Ricerche (IFN-CNR), Milan, Italy; 6 Christian Doppler Laboratory for Photonic Quantum Computer, Faculty of Physics, University of Vienna, Vienna, Austria; 7 Institute for Quantum Optics and Quantum Information (IQOQI) Vienna, Austrian Academy of Sciences, Vienna, Austria

**Keywords:** Single photons and quantum effects, Quantum information

## Abstract

Recently, machine learning has had remarkable impact in scientific to everyday-life applications. However, complex tasks often require the consumption of unfeasible amounts of energy and computational power. Quantum computation may lower such requirements, although it is unclear whether enhancements are reachable with current technologies. Here we demonstrate a kernel method on a photonic integrated processor to perform a binary classification task. We show that our protocol outperforms state-of-the-art kernel methods such as gaussian and neural tangent kernels by exploiting quantum interference, and provides further improvements in accuracy by offering single-photon coherence. Our scheme does not require entangling gates and can modify the system dimension through additional modes and injected photons. This result gives access to more efficient algorithms and to formulating tasks where quantum effects improve standard methods.

## Abstract (web summary)

A quantum kernel estimation by which feature data points are evaluated through the unitary evolution of two-boson Fock states is experimentally demonstrated on a photonic integrated processor. This model provides enhanced accuracy with respect to commonly used classical methods for several classification tasks.

## Main

The past decades have witnessed a swift development of technologies based on quantum mechanical phenomena, opening up new perspectives in a wide spectrum of applications. These range from the realization of a global-scale quantum communication network—the quantum internet—to the simulation of quantum systems and to quantum computing^1,2^. In particular, the interest towards quantum computing has been fuelled by some milestone discoveries, such as Shor’s^3^ factorization and Grover’s search algorithm^4^, which have indicated that quantum processors can outperform their classical counterparts. However, a clear advantage of quantum computation has been experimentally demonstrated only recently and on different computational tasks, such as boson sampling^5–7^ and random circuit sampling^8^, which do not have clear practical applications.

Given these premises, our goal is to investigate the tasks in which quantum computing can enhance the operation of classical computers for practically relevant tasks. Moreover, the question is whether this can be achieved for problems that are now within the reach of state-of-art technology, where only noisy intermediate-scale quantum computers are available^9^. In this context, a flurry of interest has been devoted to the open question of whether the new paradigm of quantum computing can have an impact on machine learning^10^, which has revolutionized classical computation, granting new possibilities and changing our everyday lives, from email filtering to artificial intelligence. The two main directions that have been investigated so far are, on the one hand, whether quantum computation could improve the efficiency of the learning process, enabling us to find better optima with the need of a lower number of inquiries^11–14^ and, on the other, how quantum behaviours can enhance the expressivity of the input encoding, exploiting correlations between variables that are hard to reproduce through classical computation^15,16^.

In this context, a straightforward application of quantum computing on kernel models has become evident. Kernel methods are widely used tools in machine learning^17,18^ that base their functioning on the fact that patterns for data points, which are hard to recognize in their original space, can become easy to identify once mapped nonlinearly to a feature space. When the suitable mapping is performed, it is possible to identify the hyperplane that best separates the classes of feature data points, through a support vector machine^19^ (SVM), according to the inner product of the mapped data. Let us note that the only part of the model that is trained is the SVM, which is efficient, once the inner products are available. Hence, an interesting question is whether using a quantum apparatus to perform the data mapping and evaluate the inner products can enhance the overall performance of the algorithm, benefitting from feature maps exploiting the evolution of quantum systems and outsourcing the hardest part of the computation to the quantum hardware. This question was theoretically and rigorously answered in the affirmative by Liu and colleagues^20^, although implementation of the proposed task is out of reach for state-of-the-art technologies. Hence, it is still an open question, whether some improvement in the performance of such a method can be achieved through a quantum feature map, for tasks that can be implemented on current quantum platforms. In this context, a moderately sized quantum feature space can be proved to be more suitable for preserving the similarity among data that belong to the same class. The reason for this is that the advantage of a high-dimensional feature space, enabling the expressivity of the model to be improved, is cancelled out by the dimension increase per se that typically decreases the overlap between different states, such that they become almost orthogonal^21^. This is due to the fact that the expected overlap value of two random states amounts to the inverse of the space dimension and, in high-dimensional metric spaces, almost all probability measures concentrate around the mean or median of a function (concentration of measure^22^). The effect is then that the inner products among the feature data points will tend to vanish and a higher precision will be required to properly measure them. This ultimately leads to the so-called exponential concentration^23,24^, where it is not possible to capture the information about the similarities between states, making the model—to all intents and purposes—useless for any type of classification.

**Fig. 1:**
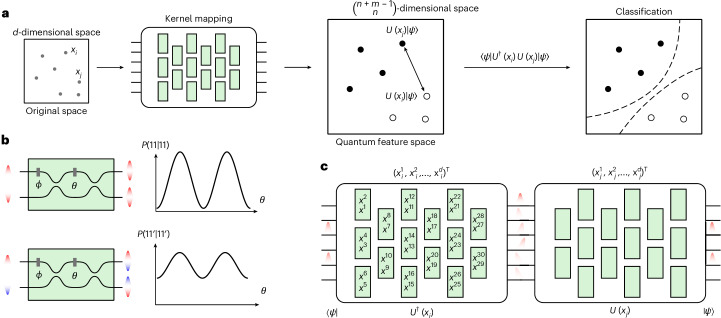
Photonic quantum kernel estimation. **a**, The photonic quantum kernel maps each data point *x*_*i*_ to be classified from a *d*-dimensional space into a quantum state ${\left\vert \Phi \right\rangle }_{i}$, living in a Hilbert feature space. In detail, the classical data *x*_*i*_ are encoded into a unitary evolution *U*(*x*_*i*_) applied on a fixed input state $\left\vert \psi \right\rangle$. This implies ${\left\vert \Phi \right\rangle }_{i}=U({x}_{i})\left\vert \psi \right\rangle$. After mapping all of the data points in the dataset, from the inner pairwise products, given by $\langle \psi| U(x_i){^{\dag}} U(x_j)|\psi\rangle$, we perform the classification, finding the hyperplane that best separates the classes, that is, through an SVM, according to equation (1). **b**, Pairs of indistinguishable photons (top) and distinguishable photons (bottom) show different behaviour when injected into a Mach–Zehnder interferometer (MZI), with external phase *Φ* and internal phase *θ*. Here, input states 11 and 11′ indicate, respectively, two indistinguishable and distinguishable photons being injected into the circuit and being detected at the output modes. *P*(*o*_1_, *o*_2_| *i*_1_, *i*_2_) is the probability of registering *o*_1_ and *o*_2_ photons, respectively, on the first and second output of the Mach–Zehnder interferometer, conditioned on having *i*_1_ and *i*_2_ photons on the input modes. **c**, Estimation of the inner product of two data points *x*_*i*_ and *x*_*j*_ by encoding them in two unitaries *U*(*x*_*i*_) and *U*(*x*_*j*_). The inner product $\left\langle {\phi }_{j}| {\phi }_{i}\right\rangle$ amounts to $\left\langle \psi | {U}^{\dagger }({x}_{i})U({x}_{j})| \psi \right\rangle$. This is equivalent to projecting the evolved state ${U}^{\dagger }({x}_{j})U({x}_{i})| \psi \rangle$ onto $| \psi \rangle$. Each green-shaded box represents a programmable MZI with two free parameters (namely, a beamsplitter with tunable reflectivity and phase), as shown in **b**.

Here we investigate this topic and experimentally demonstrate a quantum kernel estimation, where feature data points are evaluated through the unitary evolution of two-boson Fock states (Fig. 1). Such encoding, even for relatively small dimensions, proves suitable as it enables high classification accuracies to be achieved.

Furthermore, we show that, for given tasks, this algorithm leads to an enhancement in the performance of quantum kernels with respect to their classical counterparts. These tasks are selected by maximizing the so-called geometric difference, which measures the separation in performance between a pair of kernels^21^. In particular, we separate between quantum and classical kernels, that is, photonic kernels that do or do not exhibit quantum interference, respectively.

In our implementation, we exploit a photonic platform based on an integrated photonic processor^25^ where we inject two-boson Fock states to map the data to be classified (Fig. 1a). To estimate quantum and classical kernels, we inject indistinguishable and distinguishable photons, respectively. This photonic platform is particularly suitable for this task, as it enables us to encode and manipulate our input data with high fidelity.

To benchmark our enhanced performance, we compare classification accuracies between photonic kernels and state-of-the-art classical computational kernels. These include the standard gaussian kernels^17,18^ and the recently introduced neural tangent kernels^26^, which simulate gradient descent over infinitely-wide neural networks. Our results show that photonic kernels outperform classical methods and that the accuracies are further enhanced in kernels displaying quantum interference.

## Photonic quantum kernel estimation

The core of a kernel method is constituted by a function that maps nonlinearly a set of *N* data points into a feature space. In its simplest version, these data points belong to two classes, and the final goal of the algorithm is to separate them accordingly, using only a linear model, that is, a hyperplane. This is feasible as the map, being nonlinear, will change the relative position between the data points, making the dataset (in general) easier to classify. For this reason, an SVM can effectively find a hyperplane that separates the two classes, with a high accuracy. From a more mathematical perspective, we can describe the map as a function that sends *N* input data points *x*_*i*_, on which we wish to perform binary classification, from a space ${\mathcal{X}}\subseteq {{\mathbb{R}}}^{d}$ into a Hilbert space ${\mathcal{H}}$. Here, *d* is the dimension of each data point. This is done through a feature map $\Phi :{\mathcal{X}}\to {\mathcal{H}}$. Then, an SVM can be used to produce a prediction function ${f}_{K} :{\mathcal{X}}\to {\mathbb{R}}$ as *f*_*K*_(*x*) = ∑_*i*_*α*_*i*_*K*(*x*,*x*_*i*_), where these *α*_*i*_ coefficients are obtained by solving a linear optimization problem. The inputs of the optimization are the labels *y*, and the matrix obtained by computing the pairwise distances between data points is ${K}_{i,\;j}=K({x}_{i},{x}_{\!j})={| \langle \Phi ({x}_{i})| \Phi ({x}_{\!j})\rangle | }^{2}$, the so-called Gram matrix (see Supplementary Note 1 for further information).

In this work, we implement a quantum version of the kernel method, in which the aforementioned pairwise distances between data points, which belong to a class *y* taking values +1 or −1, are estimated by sampling from the output probability distribution that arises from the unitary evolution of a Fock input state. This process is depicted in Fig. 1a. Therefore, our feature map plugs the data that need to be classified into the free parameters defining a unitary evolution applied to a fixed Fock state of dimension *m* and whose sum of occupational numbers is *n*: $x\mapsto | \Phi (x)\rangle ={U}_{x}| \psi \rangle$. Here, $\left\vert \psi \right\rangle$ is the encoding state which is free to choose. Then, as shown in Fig. 1c, the pairwise inner products of the feature points are experimentally evaluated, as $| \langle \psi | U{({x}_{i})}^{\dagger }U({x}_{j})| \psi \rangle {| }^{2}$. Such unitaries can be effectively implemented by a programmable photonic circuit that consists of an array of Mach–Zehnder interferometers^27^. Hence, the dimension of the feature Hilbert space ${\mathcal{H}}$ will be $$\left(\begin{array}{c}n+m-1\\ n\end{array}\right)$$. At this point, the SVM finds the hyperplane separating the training data points through the aforementioned optimization process^28^, and the binary classification of unknown points *x* is given by the following relation:

1 $$y={\rm{sgn}}\left(\mathop{\sum }\limits_{i=1}^{N}{\alpha }_{i}\;{y}_{i}K(x,{x}_{i})\right)$$

where *α*_*i*_ are the coefficients optimized in the training process and *y*_*i*_ is the class of the *i*th point in the training. This model is defined implicitly, as the labels are assigned by weighted inner products of the encoded data points^29–34^.

If the Fock state contains indistinguishable bosons, they will exhibit quantum interference, as shown in Fig. 1b. In this case, the output probability distribution is given by the permanents of submatrices of the matrix that represents the unitary evolution of the input^35^. More specifically, considering an input configuration *s*, the probability of detecting the output configuration *t* is given by $| {\rm{Per}}{U}_{s,t}{| }^{2}/{\Pi }_{i}^{m}{s}_{i}!{\Pi }_{i}^{m}{t}_{i}!$. Here, Per( ⋅ ) denotes the permanent matrix operation, Π stands for the product notation, *s*_*i*_ and *t*_*i*_ are the occupational numbers at the *i*th mode and *U*_*s*,*t*_ is the submatrix obtained by selecting the rows/columns corresponding to the occupied modes of the input/output Fock states. On the other hand, if the bosons are distinguishable, they will not exhibit quantum interference. In this case, the probability will amount to ${\rm{Per}}| {U}_{s,t}{| }^{2}/{\Pi }_{i}^{m}{s}_{i}!{\Pi }_{i}^{m}{t}_{i}!$.

In the following, we will refer to a kernel implemented with indistinguishable bosons as a quantum kernel:

2 $${K}_{{\mathrm{Q}}}({x}_{i},{x}_{j})=| {\rm{Per}}{U}_{\psi }({x}_{i},{x}_{j}){| }^{2}/{N}^{{\prime} },$$

and with distinguishable ones as a classical kernel:

3 $${K}_{{\mathrm{C}}}({x}_{i},{x}_{j})={\rm{Per}}| {U}_{\psi }({x}_{i},{x}_{j}){| }^{2}/{N}^{{\prime} }.$$

Here, *U*_*ψ*_(*x*_*i*_,*x*_*j*_) is the matrix defined by data points *x*_*i*_ and *x*_*j*_ and the selected input state *ψ*, and *N*′ is the denominator $\Pi_i^m s_i!\Pi_i^m t_i!$ given by the occupational numbers of *ψ*. As long as there is at most one photon in each mode of *ψ*, *N*′ = 1.

## Classification task

**Fig. 2:**
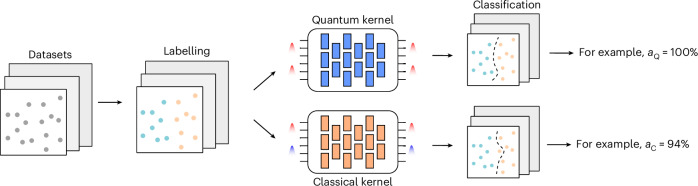
Classification tasks for photonic kernel methods. The datasets are randomly generated and consist of *d*-dimensional vectors, with entries between 0 and 1. Then, we randomly assign labels to each point as belonging to class +1 or −1 and we test the ability of our photonic kernels, displaying and not displaying quantum interference (indicated, respectively, as quantum kernel and classical kernel), to correctly classify the data. This is quantified by the accuracy of our models, which we indicate as *a*_Q_ and *a*_C_.

At this point, we need to select a classification task that will benefit from the described model. Our strategy will therefore consist, first, of generating random data points and, second, selecting the proper labelling to boost the performance of the quantum kernel (*K*_Q_) with respect to the classical kernel (*K*_C_), as depicted in Fig. 2. Thus, we need a quantifier that estimates the difference in the performance of the two models and, to this end, we select the model complexity, which is defined as *s*_*K*_(*y*) = *y*^T^*Ky*, where *K* is the kernel and *y* the labelling. This quantity is proportional to the upper bound on the classification error of a given model, and it amounts to the number of features required to make accurate predictions (Supplementary Note 2). Hence, the potential advantage given by *K*_Q_, with respect to *K*_C_, is based on finding the largest possible separation between the two model complexities^21^. Therefore, our goal is to find the optimal labelling *y*, such that

4 $$y=\arg \mathop{\min }\limits_{y\in {{\mathbb{R}}}^{d}}\left(\frac{{s}_{{K}_{{\mathrm{Q}}}}(\;y)}{{s}_{{K}_{{\mathrm{C}}}}(\;y)}\right).$$

Now, let us consider the following asymmetric distance *g*, denoted as geometric difference, between our two kernels, *K*_Q_ and *K*_C_:

5 $${g}_{{\mathrm{CQ}}}=\sqrt{{\left\Vert\sqrt{{K}_{{\mathrm{Q}}}}{\left({K}_{{\mathrm{C}}}\right)}^{-1}\sqrt{{K}_{{\mathrm{Q}}}}\right\Vert}_{\infty }},$$

where ∥ ⋅ ∥_*∞*_ denotes the spectral norm. It can be shown that the following inequality holds^21^:

6 $${s}_{{K}_{{\mathrm{C}}}}(\;y) \le {g}_{{\mathrm{CQ}}}^{2}{s}_{{K}_{{\mathrm{Q}}}}(\;y).$$

Hence, if we maximize the geometric difference, we will have at least one task that saturates inequality (6), for which the model complexity will be higher for the classical kernel with respect to the quantum kernel. This indicates that the performance of *K*_Q_ will be competitive or better than *K*_C_.

We can now use equation (5) to generate the classification task that, given two kernels *K*_Q_ and *K*_C_ and a set of data points {*x*_*i*_}, produces the labels {*y*_*i*_} that maximise the difference in prediction error bound. This can be done through the following procedure: (1) evaluate the Gram matrices *K*_Q_ and *K*_C_ over a set of non-labelled data points {*x*_*i*_}; (2) compute the positive definite matrix $M=\sqrt{{K}_{{\mathrm{Q}}}}{\left({K}_{{\mathrm{C}}}\right)}^{-1}\sqrt{{K}_{{\mathrm{Q}}}}$; (3) compute the eigenvalues and eigenvectors of *M* via spectral decomposition; (4) find the maximum eigenvalue *g* and its corresponding eigenvector *v*; and (5) assign the labels $y=\sqrt{{K}_{{\mathrm{Q}}}}v$. From a practical point of view, we start with the two aforementioned kernels, *K*_C_ and *K*_Q_, and then, by maximizing the geometric difference, we find the tasks for which the latter brings an enhanced accuracy of the classification. For more details regarding the algorithm to define the classification task, see Supplementary Note 4. Let us note that the implemented tasks constitute instances of problems that can be naturally implemented with high accuracy on our quantum platform. As such, they constitute a first stepping stone towards the identification of practical tasks for which quantum machine learning can enhance the performance of classical models.

## Experiment

**Fig. 3:**
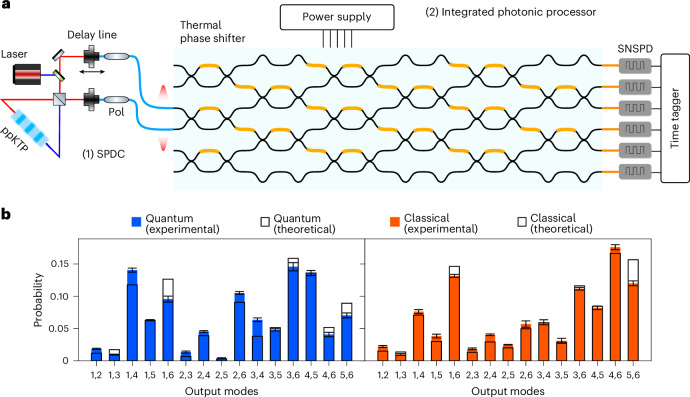
Implementation of photonic quantum kernel estimation. **a**, The experimental set-up consists of two parts: (1) the off-chip single-photon source and (2) the programmable integrated photonic processor. The frequency-degenerate photons are generated using a type-II SPDC source. Afterwards, the two photons are made indistinguishable in their polarization and arrival time. To enhance the quality of interference, two in-fiber polarizers (Pol) are placed at the two arms of the source. Then, we inject the generated photons in two modes of an integrated photonic processor with six input/output modes^25^. Detection is performed by SNSPDs. The degree of indistinguishability can then be tuned through a delay line, changing their relative temporal delay. **b**, Probability distribution of photon detection events. We show two instances of the experimental photon detection probability compared with the theoretical calculation. The quantum and classical kernel measurements are obtained, respectively, by injecting two indistinguishable and distinguishable photons into the third and fourth modes of the circuits, that is, $| 0,0,1,1,0,0\rangle$. The *x* axis shows all of the circuit channels that output two photons simultaneously. Thus, all 15 possible photon detection configurations are accessible. The error bars shown in the plot were evaluated through a Monte Carlo simulation, considering an underlying Poissonian distribution of the counts, and represent one standard deviation. The measurements were performed with an integration time of 5 s, at a detection rate of 10 kHz.

Our experimental set-up consists of two parts, a single-photon source generating the input states and a programmable integrated photonic processor, as depicted in Fig. 3a. First, to generate the input state, we use a type-II spontaneous parametric down-conversion (SPDC) source, which generates frequency-degenerate single-photon pairs at 1,546 nm in a periodically poled K-titanyl phosphate (ppKTP) crystal. The two photons are then made indistinguishable in their polarization and arrival time, respectively, via wave retarders and a delay line, which we also use to tune the degree of indistinguishability of the generated photons.

For the implementation of photonic kernels, which map our input data to a feature space, we require an apparatus that is able to perform arbitrary unitary transformations on a given input state. As mentioned above, our feature map sends each data point *x*_*i*_ onto the state resulting from the evolution *U*(*x*_*i*_) of a fixed input Fock state $| \psi \rangle$. Then, for application of the SVM, which finds the best hyperplane separating the data, we need to evaluate the inner products between all of the points *x*_*i*_, *x*_*j*_ in the feature space, which amounts to $\langle \psi | U{({x}_{i})}^{\dagger }U({x}_{j})| \psi \rangle$. This implies that, if we take $| \psi \rangle$ as a Fock state of *n* photons over *m* modes, the inner product $\langle \Phi({x}_{i})|\Phi({x}_{j})\rangle$ is given by projecting the evolved state $U{({x}_{i})}^{\dagger }U({x}_{j})\left\vert \psi \right\rangle$ onto $\left\vert \psi \right\rangle$.

To this end, we use an integrated photonic processor^25^ on a borosilicate glass substrate, in which optical waveguides are inscribed through femtosecond laser writing^36^. The circuit features six input/output modes^27^ (as depicted in Fig. 3a) where each interferometer is equipped with two thermal phase shifters^37^, to provide tunable reflectivity and phase. Such an arrangement enables any unitary transformation to be performed on the input photon states. Given this property, our device is also referred to as a universal photonic processor. The design, fabrication and calibration of the integrated photonic circuit are described in ref. ^25^.

Specifically, the data were encoded in the values of the phase shifts as follows: ${x}_{i}=({x}_{i}^{1},{x}_{i}^{2},\ldots ,{x}_{i}^{30})\to {\theta }_{i}=(2\uppi {x}_{i}^{1},2\uppi {x}_{i}^{2},\ldots ,2\uppi {x}_{i}^{30})$, where *θ*_*i*_ are the phase shifts introduced by the phase shifters of a universal interferometer. Let us note that this encoding has the remarkable advantage that no extra processing is required on the input data, as they are plugged directly into the optical circuit parameters. Furthermore, in principle, we would need a sequence of two of such circuits (as in the scheme of Fig. 1c), to first implement *U*^†^(*x*_*i*_) and then *U*(*x*_*j*_) on our inputs. However, in our implementation, we adopt only one universal circuit and directly implement the unitary corresponding to the product *U*(*x*_*i*_)^†^*U*(*x*_*j*_). This reduces the experimental complexity and the circuit propagation losses.

At the output, detection is performed using superconducting nanowire single-photon detectors (SNSPDs), where we post-select the output events to those featuring two detector clicks (coincidence counts (CC)), that is, collision-free events (Supplementary Note 3). Thus, the elements of the Gram matrix of a given kernel can be estimated from the coincidence counting $K({x}_{i},{x}_{j})={{\rm{CC}}}_{\psi }^{ij}/{\sum }_{1\le l < m\le 6}{{\rm{CC}}}_{lm}^{ij}$. Here, ${{\rm{CC}}}_{lm}^{ij}$ is the number of registered coincidence counts between channels *l* and *m*, when the implemented unitary is *U*^†^(*x*_*j*_)*U*(*x*_*i*_) and *ψ* indicates the occupied modes of input state $| \psi \left.\right\rangle$. To test the role of quantum interference in the accuracy of the classification, we tune the indistinguishability of the two photons by changing their relative temporal delay. An instance of the probability distribution of the same unitary is shown in Fig. 3b. The optimal classification task is chosen for each dataset according to the algorithm as explained in the previous section.

## Results

We tested the performance of two photonic kernels in several different configurations. First, we considered two different inputs, $\left\vert 1,1,0,0,0,0\right\rangle$ and $\left\vert 0,0,1,1,0,0\right\rangle$. This amounts to either injecting the photons into the first two modes or the central two modes. Second, we were able to control the indistinguishability of the bosons, which allows us to implement, investigate a compare a quantum kernel with its related classical kernel. This is achieved by tuning the relative temporal delay of the propagating photons. Consequently, at the detection, it is possible to (partially) distinguish which photon—out of the two input photons—is detected, by checking their arrival time. Perfect distinguishability is obtained when the temporal delay is longer than the coherence length of the photons. During the whole measurement, the maximal achieved indistinguishability between the photons was 0.9720 ± 0.0044, estimated via on-chip Hong–Ou–Mandel interference^38^.

For both input states, we fixed the encoding of each data point and varied the datasets to have four different sizes: 40, 60, 80 and 100. We used the set-up depicted in Fig. 3a to implement all pairwise products between the unitaries *U*(*x*_*i*_)^†^*U*(*x*_*j*_). Hence, $| \langle \psi | U{({x}_{i})}^{\dagger }U({x}_{j})| \psi \rangle {| }^{2}$ is given by the probability of detecting the photons on the same modes from which they were injected. The rate of total post-selected coincidence counts amounts to 10 kHz, and the measured probability distribution was averaged over 5 s for each unitary configuration. Let us point out that restricting to collision-free events is unlikely to bring to a decay in the rate of detected samples when considering larger systems, as the bunching events become in general more rare for higher-dimensional Hilbert spaces, in the absence of particular symmetry patterns in the matrix describing the unitary evolution of the input^5,35^.

For each size *N*, we performed *N*(*N* − 1)/2 unitaries to compute the inner products. The distance between the unitaries can be experimentally realized and the target ones can be estimated as ${\sum }_{i}\sqrt{{P}_{i}^{{\rm{theo}}}{P}_{i}^{\exp }}$, where ${P}_{i}^{\exp }$ is the experimental detection frequency for the *i*th output configuration, whereas ${P}_{i}^{{\rm{theo}}}$ is the detection frequency for the *i*th output configuration estimated on the basis of theory^35^. For the quantum kernel and classical kernel, the mean fidelity of all datasets is 0.9816 ± 0.0148 and 0.9934 ± 0.0048, respectively. For each dataset, we use two-thirds of the data points for training the SVM, which yields the coefficients mentioned in equation (1). The remaining one-third as the test dataset can be used to predict the classification accuracy. The accuracy is defined as the percentage of correctly classified points out of the total size of the test set. Let us note that values lower than 0.5 indicate that the model was not able to learn the features of the training set and generalize to unknown data.

**Fig. 4:**
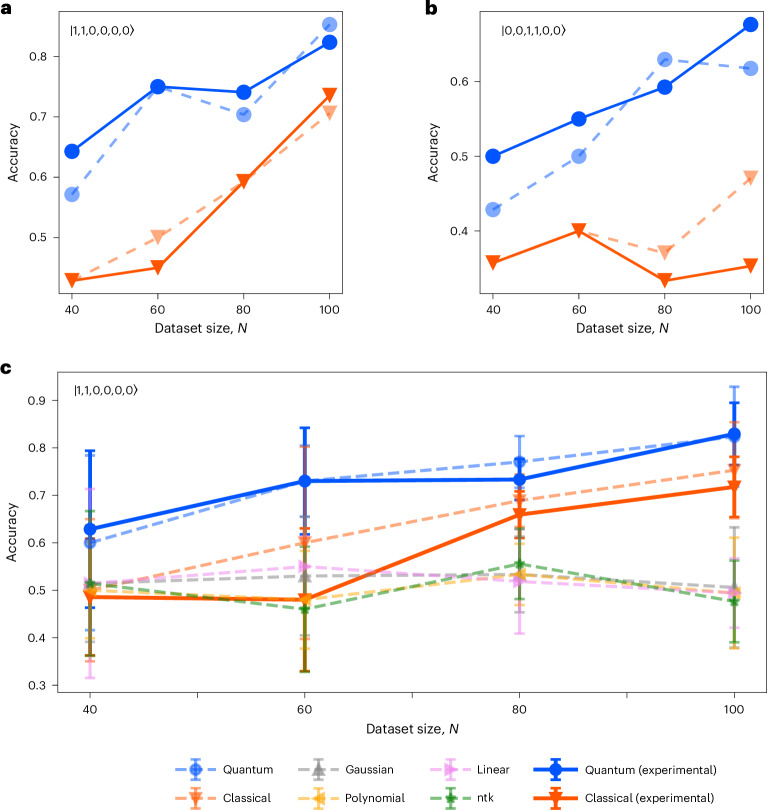
Experimental classification accuracies. **a**,**b**, We tested our method on datasets of different sizes (40, 60, 80 and 100) and for two different input states of $| 1,1,0,0,0,0\rangle$ (**a**) and $| 0,0,1,1,0,0 \rangle$ (**b**). For each dataset, two-thirds of the data points were used for training the SVM, and the remaining one-third were used for testing. **c**, The average classification accuracies on five different sets for the quantum kernel (blue) and the classical kernel (orange), along with the following other computational kernels: gaussian (grey), ntk (green), polynomial (yellow) and linear (purple). The dashed lines indicate the results of numerical simulations, whereas the solid lines denote the experimental results. The error bars show the standard deviation of the classification accuracies on five random generated datasets classified by all of the kernels.

In Fig. 4a,b, we show the test accuracies obtained by injecting two input states for four different dataset sizes, where the quantum kernel performs better than the classical kernel in both experiments. In Fig. 4c, we report the average test accuracy obtained for five different datasets of the same size in addition to varying the dataset size from 40 to 100. Moreover, the results obtained with the quantum kernel (blue) and the classical kernel (orange) are compared with the following numerical kernels: the neural tangent kernel (ntk, green)^26^, gaussian kernel (grey), polynomial kernel (yellow) and linear kernel (purple)^17,18^. Here, the neural tangent kernel adopts an infinite-width neural network to classify the data optimized by gradient descent—see Supplementary Note 5 for more details. The dashed lines indicate the results of numerical simulations, whereas the solid lines indicate experimental results. Although the task is built comparing only the performance of the kernels based on indistinguishable and distinguishable photons, the obtained accuracies of both are higher than the other classical kernels.

## Discussion

In this work we present the experimental demonstration of a quantum kernel estimation, based on the unitary evolution of Fock states through an integrated photonic processor. We map data into a feature space through the evolution of a fixed two-photon input state over six modes. To achieve this, we adopt an quantum processor realized via femtosecond laser writing on a borosilicate glass substrate^25^. The sampled output distribution is then fed into an SVM, performing the classification. It is of note that, although our apparatus features only tunable phase shifters and beamsplitters, such encoding produces a sufficient nonlinearity to achieve a high classification accuracy of nonlinearly separable datasets. This constitutes a difference between our method and alternative platforms, where entangling gates are typically needed^21,39^. Furthermore, in our case, it is not necessary to increase the dimension of the feature Hilbert space to achieve a good accuracy. This circumvents the typical difficulty of quantum kernels in which all data points are encoded in orthogonal states, leading to ineffective classification^21,23,24,40^. Moreover, the fact that our model is effective for small dimensions is a crucial feature, as we require an approximation of the full probability distribution that is derived from the evolution of our input state. Hence, this study is relevant for medium-sized problems, because reaching high dimensions will imply the input/output combinations to grow exponentially, along with the number of required experimental shots to reach arbitrary accuracy.

The task we implement is designed by assigning binary labels to randomly generated data points, which we encode in the phase shifts of an optical circuit. This is done by exploiting the so-called geometric difference, which selects the task for which the presence of quantum interference yields a better classification accuracy compared with cases where no interference is displayed. Although the geometric difference compares the performance of a pair of kernels (in our case kernels implemented with indistinguishable/distinguishable bosons), both photonic kernels performed significantly better for the selected tasks than commonly used kernels, not only gaussian, polynomial and linear kernels but also the neural tangent kernel. This improvement in the performance that is seen for both photonic kernels is probably due to the fact that both the statistics have a similar dependence on the unitary evolution matrix, which is based on the permanent of submatrices (equations (2) and (3)), and are both complex enough to successfully tackle the selected tasks. In summary, our results indicate that a photonic kernel estimation can enhance the performance—even for medium-sized problems—and is reachable by current quantum technologies. Moreover, using distinguishable bosons to have a (smaller) performance enhancement represents an intriguing possibility as it can prove convenient to reduce the impact of photon losses on the experimental time required to collect significant statistics.

Let us highlight that, although the interference of two photons in a six-mode unitary matrix (or more in general for medium-sized problems) can be estimated using classical computers, this does not affect the features of our protocol. First, this is because the interest in investigating the potentialities and limitations of a new computational method, such as our proposed kernel, goes beyond the platform (quantum or classical) where it can be implemented, especially considering its particular suitability for given tasks. This is also the reason why we do not use our photonic platform to reproduce classical kernels, as done in refs. ^30,41^, but focus on investigating those given by the natural evolution of bosons through a quantum circuit. Second, the approximation of permanents through classical algorithms, for example, the algorithm of Gurvits^42^, has a slightly worse performance when compared with direct sampling from an optical circuit, which naturally implements the studied kernel, for the case of inputs featuring product states^43^. In particular, the first scales as *O*(*n*^2^/*e*^2^), where *n* is the number of photons, *e* is the required precision and *O* stands for the asymptotic time complexity of the algorithms, indicating how fast their execution time grows with *n* and *e*, whereas the direct sampling as *O*(1/*e*^2^) (ref. ^44^). It is also noteworthy that the possibility of obtaining a quantum advantage on applications based on estimating expected values of unitary evolutions on linear optical circuits remains an open question^43,44^. In our case, an advantage could emerge by considering the extension of our quantum kernel ${K}_{{\mathrm{Q}}}({x}_{i},{x}_{j})=| \left\langle \right.\psi | U({x}_{i},{x}_{j})\left\vert \psi \right\rangle {| }^{2}$ for entangled input states *ψ*, although there is no rigorous proof of that yet. Third, this protocol sheds light on alternative computational models, exploiting optical computation. This may be of particular importance when considering difficulties related to energy consumption, as it has been proved that partially optical networks can reduce energy requirements with respect to electronic ones^45^.

Despite being overshadowed by deep neural networks, kernels are still widely used in a large number of tasks due to their simplicity and ability to learn from small datasets^46,47^. Indeed, they have seen a recent revival in classical machine learning with the introduction of neural tangent kernels^26^ and their use in the study of state-of-the-art neural network architectures such as transformers^48^. Another recent trend consists of merging neural networks and kernels, where notable examples are attention modules in natural language processing and Hopfield layers^49^. Also from the quantum computing point of view, kernels have recently gained a flurry of interest, both from the theoretical as well as from the experimental side^23,41,50,51^. For the latter, it has been proved that the evolution of (photonic) quantum states through a circuit is complex enough to tackle image recognition tasks, both for simple toy models^41,51^ and satellite images^50^. Our method can therefore find a wide range of promising near-term applications in tasks such as information retrieval, natural language processing and medical image classification^52–55^, where kernels have been proposed as a keystone^56^.

In particular, an interesting approach involves pre-training a small (single-qubit) system to find the optimal encoding of classical data, which is then classified through a kernel method featuring a larger system, as proposed in ref. ^51^. This could be particularly beneficial on our photonic platform as the pre-training could be performed through a classical light input over two spatial modes. This would imply outsourcing the computationally hardest part of the protocol to a fully optical platform, possibly bringing a significant reduction in energy consumption. Our experimental results also open the door to hybrid methods in which photonic processors are used to enhance the performance of standard machine learning methods. They also bring forward investigations on the nonlinearities that can be achieved through photonic platforms^57,58^, in particular, for neuromorphic computation models, such as reservoir computing^59,60^. In addition, we envisage the combination of this kind of nonlinearity with that brought by the implementation of feedback loops, as in the case of a quantum memristor^58^ and the exploitation of quantum interference in the implementation of feature maps.

## Methods

The two-photon input states are generated by a type-II SPDC source, which generates frequency-degenerate single-photon pairs at 1,546 nm via a 3-cm-long ppKTP crystal. Afterwards, the two photons are made indistinguishable in their polarization (which is rotated through paddles) and in arrival time (through a delay line, which we also use to tune the degree of indistinguishability of the generated photons).

We then inject these photons into two selected modes of an integrated photonic processor with six input/output modes. This circuit features 27 thermal phase shifters and its architecture is universal, enabling us to implement arbitrary unitary evolution on any input Fock state. Compared with the original Clements architecture, the first three external phase shifters are not implemented as they will not affect the probability distribution, in the case of inputs not featuring coherent superpositions of Fock states. To apply accurate phases independently, each channel is supplied by a current source to avoid electrical crosstalk (4 × Qontrol Q8iv driver modules, 16-bit DAC). At the end, detection is performed by SNSPDs housed in a 1K cryostat. We post-select the detected events to the cases in which two detectors click simultaneously in a temporal window of 1 ns. A time tagger with a 15.63 ps resolution is used to process the real-time coincidence counting for all 15 post-selection patterns. The total coincidence counting is about 10 kHz, which varies due to the pump laser and the detection efficiency. For each unitary configuration, we integrate for 5 s to estimate the probability distribution over 15 coincidence patterns.

## Online content

Any methods, additional references, Nature Portfolio reporting summaries, source data, extended data, supplementary information, acknowledgements, peer review information; details of author contributions and competing interests; and statements of data and code availability are available at 10.1038/s41566-025-01682-5.

## Supplementary information

Supplementary Information Supplementary Notes 1–6, Figs. 1–8 and refs. 1–6.

## Data Availability

The data that support the findings of this study and detailed explanations of the datasets are available from the project page via GitHub at https://github.com/dapingQ/PhoQuKs.

## Code availability

The code scripts used in this study are available from the project page via GitHub at https://github.com/dapingQ/PhoQuKs.

## References

[1] Wehner, S., Elkouss, D. & Hanson, R. Quantum internet: a vision for the road ahead. *Science* **362**, eaam9288 (2018). 30337383 10.1126/science.aam9288

[2] Georgescu, I. M., Ashhab, S. & Nori, F. Quantum simulation. *Rev. Mod. Phys.* **86**, 153–185 (2014).

[3] Shor, P. W. Algorithms for quantum computation: discrete logarithms and factoring. In *Proc. 35th Annual Symposium on Foundations of Computer Science* (ed. Johnson, D. S.) 124–134 (IEEE, 1994).

[4] Grover, L. K. A fast quantum mechanical algorithm for database search. In *STOC ’96: Proc. 28th Annual ACM Symposium on Theory of Computing* (ed. Miller, G. L.) 212–219 (Association for Computing Machinery, 1996).

[5] Aaronson, S. & Arkhipov, A. The computational complexity of linear optics. In *STOC* ’*11: Proc. 43rd Annual ACM Symposium on Theory of Computing* (eds Fortnow, L. & Vadhan, S. P.) 333–342 (Association for Computing Machinery, 2011).

[6] Zhong, H.-S. et al. Quantum computational advantage using photons. *Science* **370**, 1460–1463 (2020). 33273064 10.1126/science.abe8770

[7] Madsen, L. S. et al. Quantum computational advantage with a programmable photonic processor. *Nature* **606**, 75–81 (2022). 35650354 10.1038/s41586-022-04725-x PMC9159949

[8] Arute, F. et al. Quantum supremacy using a programmable superconducting processor. *Nature* **574**, 505–510 (2019). 31645734 10.1038/s41586-019-1666-5

[9] Preskill, J. Quantum computing in the NISQ era and beyond. *Quantum* **2**, 79 (2018).

[10] Dunjko, V. & Briegel, H. J. Machine learning & artificial intelligence in the quantum domain: a review of recent progress. *Rep. Prog. Phys.* **81**, 074001 (2018). 29504942 10.1088/1361-6633/aab406

[11] Neven, H., Denchev, V. S., Rose, G. & Macready, W. G. Qboost: large scale classifier training withadiabatic quantum optimization. *J. Mach. Learn. Res.* **25**, 333–348 (2012).

[12] Rebentrost, P., Mohseni, M. & Lloyd, S. Quantum support vector machine for big data classification. *Phys. Rev. Lett.* **113**, 130503 (2014). 25302877 10.1103/PhysRevLett.113.130503

[13] Leifer, M. S. & Poulin, D. Quantum graphical models and belief propagation. *Ann. Phys.* **323**, 1899–1946 (2008).

[14] Saggio, V. et al. Experimental quantum speed-up in reinforcement learning agents. *Nature* **591**, 229–233 (2021). 33692560 10.1038/s41586-021-03242-7 PMC7612051

[15] Boixo, S. et al. Characterizing quantum supremacy in near-term devices. *Nat. Phys.* **14**, 595–600 (2018).

[16] Gan, B. Y., Leykam, D. & Angelakis, D. G. Fock state-enhanced expressivity of quantum machine learning models. *EPJ Quantum Technol.* **9**, 16 (2022).

[17] Shawe-Taylor, J. & Cristianini, N. *Kernel Methods for Pattern Analysis* (Cambridge Univ. Press, 2004)

[18] Hofmann, T., Schölkopf, B. & Smola, A. J. Kernel methods in machine learning. *Ann. Stat.* **36**, 1171–1220 (2008).

[19] Cortes, C. & Vapnik, V. Support-vector networks. *Mach. Learn.* **20**, 273–297 (1995).

[20] Liu, Y., Arunachalam, S. & Temme, K. A rigorous and robust quantum speed-up in supervised machine learning. *Nat. Phys.* **17**, 1013–1017 (2021).

[21] Huang, H.-Y. et al. Power of data in quantum machine learning. *Nat. Commun.* **12**, 2631 (2021). 33976136 10.1038/s41467-021-22539-9 PMC8113501

[22] Talagrand, M. Concentration of measure and isoperimetric inequalities in product spaces. *Publ. Math. Inst. Hautes Études Sci.* **81**, 73–205 (1995).

[23] Thanasilp, S., Wang, S., Cerezo, M. & Holmes, Z. Exponential concentration in quantum kernel methods. *Nat. Commun.* **15**, 5200 (2024). 38890282 10.1038/s41467-024-49287-w PMC11189509

[24] Kübler, J., Buchholz, S. & Schölkopf, B. The inductive bias of quantum kernels. In *Advances in Neural Information Processing Systems* (eds Ranzato, M. et al.) 12661–12673 (Curran Associates, 2021).

[25] Pentangelo, C. et al. High-fidelity and polarization-insensitive universal photonic processors fabricated by femtosecond laser writing. *Nanophotonics* **13**, 2259–2270 (2024). 39634510 10.1515/nanoph-2023-0636 PMC11501604

[26] Jacot, A., Gabriel, F. & Hongler, C. Neural tangent kernel: convergence and generalization in neural networks. In *Advances in Neural Information Processing Systems 31: 32nd Conference on Neural Information Processing Systems (NeurIPS 2018)* (eds Bengio, S. et al.) 8571–8580 (Neural Information Processing Systems Foundation, 2018).

[27] Clements, W. R., Humphreys, P. C., Metcalf, B. J., Kolthammer, W. S. & Walsmley, I. A. Optimal design for universal multiport interferometers. *Optica* **3**, 1460–1465 (2016).

[28] Boser, B. E., Guyon, I. M. & Vapnik, V. N. A training algorithm for optimal margin classifiers. In *COLT ’92: Proc. 5th Annual Workshop on Computational Learning Theory* (ed. Haussler, D.) 144–152 (Association for Computing Machinery, 1992).

[29] Lloyd, S., Schuld, M., Ijaz, A., Izaac, J. & Killoran, N. Quantum embeddings for machine learning. Preprint at https://arxiv.org/abs/2001.03622 (2020)

[30] Bartkiewicz, K. et al. Experimental kernel-based quantum machine learning in finite feature space. *Sci. Rep.* **10**, 12356 (2020). 32704032 10.1038/s41598-020-68911-5 PMC7378258

[31] Huang, H.-Y., Kueng, R. & Preskill, J. Information-theoretic bounds on quantum advantage in machine learning. *Phys. Rev. Lett.* **126**, 190505 (2021). 34047595 10.1103/PhysRevLett.126.190505

[32] Kusumoto, T., Mitarai, K., Fujii, K., Kitagawa, M. & Negoro, M. Experimental quantum kernel trick with nuclear spins in a solid. *NPJ Quantum Inf.* **7**, 94 (2021).

[33] Schölkopf, B. & Smola, A. J. *Learning with Kernels: Support Vector Machines, Regularization, Optimization, and Beyond* (MIT Press, 2002)

[34] Jerbi, S. et al. Quantum machine learning beyond kernel methods. *Nat. Commun.* **14**, 517 (2023). 36720861 10.1038/s41467-023-36159-y PMC9889392

[35] Tichy, M. C. Interference of identical particles from entanglement to boson-sampling. *J. Phys. B* **47**, 103001 (2014).

[36] Corrielli, G., Crespi, A. & Osellame, R. Femtosecond laser micromachining for integrated quantum photonics. *Nanophotonics* **10**, 3789–3812 (2021).

[37] Ceccarelli, F. et al. Low power reconfigurability and reduced crosstalk in integrated photonic circuits fabricated by femtosecond laser micromachining. *Laser Photon. Rev.* **14**, 2000024 (2020).

[38] Hong, C.-K., Ou, Z.-Y. & Mandel, L. Measurement of subpicosecond time intervals between two photons by interference. *Phys. Rev. Lett.* **59**, 2044–2046 (1987). 10035403 10.1103/PhysRevLett.59.2044

[39] Havlíček, V. et al. Supervised learning with quantum-enhanced feature spaces. *Nature* **567**, 209–212 (2019). 30867609 10.1038/s41586-019-0980-2

[40] Schuld, M. & Killoran, N. Quantum machine learning in feature Hilbert spaces. *Phys. Rev. Lett.* **122**, 040504 (2019). 30768345 10.1103/PhysRevLett.122.040504

[41] Hoch, F. et al. Quantum machine learning with adaptive boson sampling via post-selection. *Nat. Commun.* **16**, 902 (2025). 39837818 10.1038/s41467-025-55877-z PMC11751292

[42] Gurvits, L. On the complexity of mixed discriminants and related problems. In *Mathematical Foundations of Computer Science 2005: 30th International Symposium, MFCS 2005* (eds Jȩdrzejowicz, J. & Szepietowski, A.) 447–458 (Springer, 2005)

[43] Lim, Y. & Oh, C. Efficient classical algorithms for linear optical circuits. Preprint at https://arxiv.org/abs/2502.12882 (2025)

[44] Aaronson, S. & Hance, T. Generalizing and derandomizing Gurvits's approximation algorithm for the permanent. *Quantum Inf. Comput.* **14**, 541–559 (2014).

[45] Hamerly, R., Bernstein, L., Sludds, A., Soljačić, M. & Englund, D. Large-scale optical neural networks based on photoelectric multiplication. *Phys. Rev. X* **9**, 021032 (2019).

[46] Lee, J. et al. Finite versus infinite neural networks: an empirical study. In *Advances in Neural Information Processing Systems 33: 34th Conference on Neural Information Processing Systems (NeurIPS 2020)* (eds Larochelle, H. et al.) 15156–15172 (Neural Information Processing Systems Foundation, 2020).

[47] Radhakrishnan, A., Ruiz Luyten, M., Prasad, N. & Uhler, C. Transfer learning with kernel methods. *Nat. Commun.* **14**, 5570 (2023). 37689796 10.1038/s41467-023-41215-8 PMC10492830

[48] Tsai, Y.-H. H., Bai, S., Yamada, M., Morency, L.-P. & Salakhutdinov, R. Transformer dissection: an unified understanding for transformer’s attention via the lens of kernel. In *Proc. 2019 Conference on Empirical Methods in Natural Language Processing and the 9th International Joint Conference on Natural Language Processing (EMNLP-IJCNLP)* (eds Inui, K. et al.) 4343–4352 (Association for Computational Linguistics, 2019); 10.18653/v1/D19-1443

[49] Ramsauer, H. et al. *Proc. 9th International Conference on Learning Representations* (ICLR, 2021).

[50] Rodriguez-Grasa, P., Farzan-Rodríguez, R., Novelli, G., Ban, Y. & Sanz, M. Satellite image classification with neural quantum kernels. *Mach. Learn. Sci. Technol.* **6**, 015043 (2025).

[51] Rodriguez-Grasa, P., Ban, Y. & Sanz, M. Neural quantum kernels: training quantum kernels with quantum neural networks. Preprint at https://arxiv.org/abs/2401.04642 (2024).

[52] Wang, X., Du, Y., Luo, Y. & Tao, D. Towards understanding the power of quantum kernels in the NISQ era. *Quantum* **5**, 531 (2021).

[53] Yu, C.-H., Gao, F., Wang, Q.-L. & Wen, Q.-Y. Quantum algorithm for association rules mining. *Phys. Rev. A* **94**, 042311 (2016).

[54] Lorenz, R., Pearson, A., Meichanetzidis, K., Kartsaklis, D. & Coecke, B. QNLP in practice: running compositional models of meaning on a quantum computer. *J. Artif. Intell. Res.* **76**, 1305–1342 (2023).

[55] Landman, J. et al. Quantum methods for neural networks and application to medical image classification. *Quantum* **6**, 881 (2022).

[56] Schuld, M. & Petruccione, F. in *Machine Learning with Quantum Computers* (eds Schuld, M. & Petruccione, F.) 217–245 (Springer, 2021); 10.1007/978-3-030-83098-4_6

[57] Denis, Z., Favero, I. & Ciuti, C. Photonic kernel machine learning for ultrafast spectral analysis. *Phys. Rev. Appl.* **17**, 034077 (2022).

[58] Spagnolo, M. et al. Experimental photonic quantum memristor. *Nat. Photon.* **16**, 318–323 (2022).

[59] Govia, L. C. G., Ribeill, G. J., Rowlands, G. E. & Ohki, T. A. Nonlinear input transformations are ubiquitous in quantum reservoir computing. *Neuromorphic Comput. Eng.* **2**, 014008 (2022).

[60] Innocenti, L. et al. Potential and limitations of quantum extreme learning machines. *Commun. Phys.* **6**, 118 (2023).

