# Supplementary material for: Experimental quantum-enhanced kernel-based machine learning on a photonic processor
Source: Nat Photonics. 2025 Jun 2;19(9):1020–7. doi: 10.1038/s41566-025-01682-5 (PMC12411276; doi:10.1038/s41566-025-01682-5)
Supplement: Supplementary file 1 — Supplementary Notes 1–6, Figs. 1–8 and refs. 1–6. [file 41566_2025_1682_MOESM1_ESM.pdf]

nature photonics

Supplementary information

https://doi.org/10.1038/s41566-025-01682-5

Experimental quantum-enhanced kernel-based machine learning on a photonic processor

In the format provided by the authors and unedited

Supplementary Information
Experimental quantum-enhanced kernel-based machine learning on a photonic processor

Supplementary Note 1. SUPPORT VECTOR MACHINES AND KERNEL METHODS

For a dataset consisting of N data points with a ± 1 label, $D = \{(x_i, y_i)\}_{i=1}^{N}$, each data point is a d-length vector, $x_i = (x_i^1, x_i^2 ..., x_i^d)^T$, where $x_i \in \mathcal{X} \subseteq \mathbb{R}^d$ and x_i^j is the j-th feature of the input vector x_i. $y_i \in \mathcal{Y} = \{+1, -1\}$ is the binary label corresponding to the x_i. The classification task is to find a hyperplane $w \in \mathbb{R}^d$ and a bias parameter $b \in \mathbb{R}$, thus a linear *support vector machine* (SVM) is defined to predict the label of unknown data points x.

$$f(x) = \mathrm{sign}(w \cdot x + b) \tag{S1}$$

This is an optimization problem,

$$\min \quad \frac{1}{2}||w||^2 \tag{S2}$$

$$\text{s.t.} \quad y_i(w \cdot x_i + b) - 1 \geq 0, \quad i = 1, 2, ..., N \tag{S3}$$

and it equals to solve the dual problem,

$$\min_{\alpha} \quad \frac{1}{2}\sum_{i=1}^{N}\sum_{i=1}^{N}\alpha_i\alpha_j y_i y_j (x_i \cdot x_j) - \sum_{i=1}^{N}\alpha_i \tag{S4}$$

$$\text{s.t.} \quad \sum_{i=1}^{N}\alpha_i y_i = 0 \tag{S5}$$

$$\alpha_i \geq 0, \quad i = 1, 2, ..., N \tag{S6}$$

The solution $\{\alpha_i\}$ are obtained by solving a linear optimization problem, given by the inner products of all input vectors $x_i \cdot x_j$ and the labels y_i.

Instead, rather than the direct linear inner products, the kernel function is used to transform the input vector from the original input space to another inner product *feature space*, $\Phi : \mathcal{X} \to \mathcal{H}$. The *Gram matrix* is a symmetric matrix where each element represents the pair-wise of inner product in a feature space $K_{ij} = \Phi(x_i) \cdot \Phi(x_j)$. The prediction function based on a kernel K is given as

$$f_K(x) = \sum_{i}^{N}\alpha_i K(x, x_i) \tag{S7}$$

For some implicit kernel functions, it is easier to calculate the Gram matrices than the kernel functions. For example, if we define the feature space as *Fock space* $\mathcal{F}$, each original data point is mapped to a quantum state $x_i \mapsto |\Phi(x_i)\rangle = U(x_i)|\psi\rangle$ by a parameterized circuit $U(x_i)$ and Fock state $|\psi\rangle$.

Instead of performing quantum state tomography over every inner product, which is an expensive task considering the size of feature spaces, [1], it is straightforward to measure the outcome probability Gram matrix $K(x_i, x_j) = |\langle\Phi(x_i)|\Phi(x_j)\rangle|^2 = \left|\langle\psi|U(x_i)^\dagger U(x_j)|\psi\rangle\right|^2$.

Thus, in our experiment, the task is to estimate each element of Gram matrices for each dataset which is equivalent to estimate the outcome probability of In Fig. S7, we show an example of experimentally reconstructed Gram matrices, as well as the ones calculated by the theory.

Supplementary Note 2. GEOMETRIC DIFFERENCE

For a given kernel method and a dataset $D = \{(x_i, y_i)\}_{i=1}^{N}$, the *model complexity* of the triple (K, N, y) is given by:

$$s_K(y) = \sum_{i}\sum_{j}(K^{-1})_{ij} y_i y_j = y^T K^{-1} y \tag{S8}$$

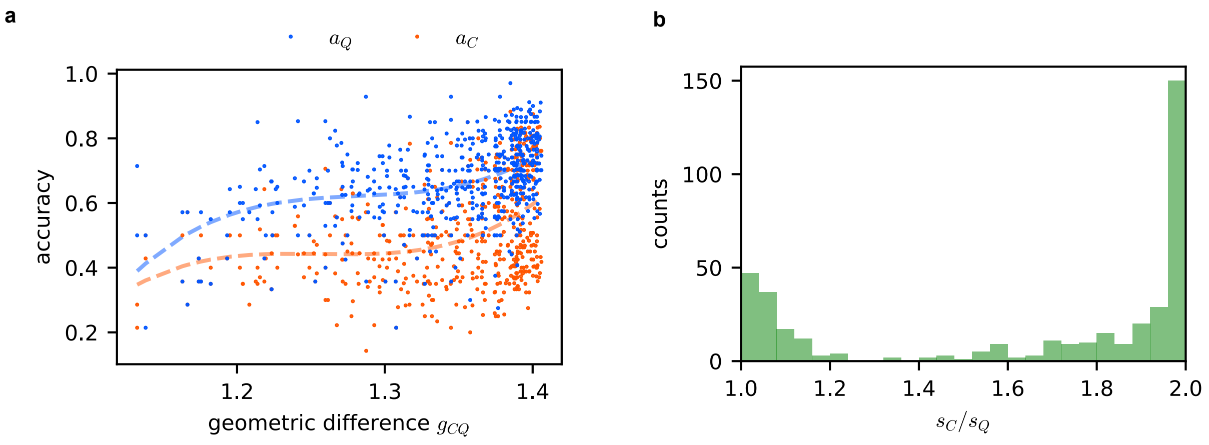

Supplementary Figure S1. **Correlation between geometric difference, classification accuracy, and model complexity.** By varying the number of data points, the width of the unitary ansatz, and the photon number, we explore the correlation between the geometric difference and the classification accuracy and model complexity. **a**. The classification accuracy of the quantum kernel and classical kernel are plotted against the geometric difference. Each pair of points (red and blue) corresponding to a given geometric different refers to the same data set. The blue dots indicate the numerical accuracy achieved on test datasets of several dimensions exploiting the quantum kernel, varying the unitary ansatz width, encoding state and dataset sizes. The red ones, instead, refer to the classical kernel. The figure shows that, on average, the geometric difference is higher than 1, which implies that the quantum kernel has higher model complexity than the classical one. **b**. We plot the histogram of s_C/s_Q, which is related to the geometric difference as in Eq. (**??**). It can be seen that the majority of the tested datasets display a higher model complexity for the classical kernel, with respect to the quantum one.

As shown in [2], this quantity can be used to bound the prediction error of a kernel method K on the dataset:

$$\mathbb{E}|f_K(x) - f(x)| \quad \leq \quad c \cdot \sqrt{\frac{s_K(y)}{N}} \tag{S9}$$

Here, $x \in \mathcal{X}$, c is a constant and N is the number of data points.

The concept *geometric difference* is used to separate the complexity of two kernels. In this work, we target two kernels K_Q and K_C, which are the transform from original space to the Hilbert space given by the evolution of indistinguishable photons and the one given by distinguishable photons, respectively. Therefore, the optimal labelling is attained by solving the optimization problem by minimizing the ratio of the model complexity s_{K_Q} to s_{K_C}:

$$y^\star = \arg\min_{y\in\mathbb{R}^N} \frac{s_{K_Q}(y)}{s_{K_C}(y)} \tag{S10}$$

The solution is $y = \sqrt{K_Q}\mathbf{v}$, where $\mathbf{v}$ is the eigenvector of matrix $\sqrt{K_Q}(K^C)^{-1}\sqrt{K_Q}$ with the eigenvalue noted as g^2_{CQ} [2]. This solution holds the inequality:

$$s_{K_C}(y) \quad \leq \quad g^2_{CQ}\, s_{K_Q}(y) \tag{S11}$$

This inequality saturates when g^2 equals the spectral norm, as follows

$$g_{CQ} = \sqrt{\left\|\sqrt{K_Q}(K_C)^{-1}\sqrt{K_Q}\right\|_\infty} \tag{S12}$$

In practice, a regularization parameter λ is used in the model complexity s_K, thus the geometric difference is alternated as

$$g_{CQ} = \sqrt{\left\|\sqrt{K_Q}(K_C+\lambda I)^{-1}\sqrt{K_Q}\right\|_\infty} \tag{S13}$$

In the following simulations, we use $\lambda = 0.02$ without loss of generality.

Based on this model, we performed several simulations of randomly generated datasets, varying the number of data points, dimensions of unitaries, and photon numbers. To explore the correlation between geometric difference and

the accuracy enhancement brought by quantum kernels, we plot test accuracies with respect to the the geometric difference in Fig. S1a. In general, a higher geometrical difference implies a better performance of the quantum kernel, with respect to the classical one. This is also reflected by Fig. S1b, where we show that, out of the sets we tested, the majority display a high ratio between the model complexity of the classical kernel and quantum one.

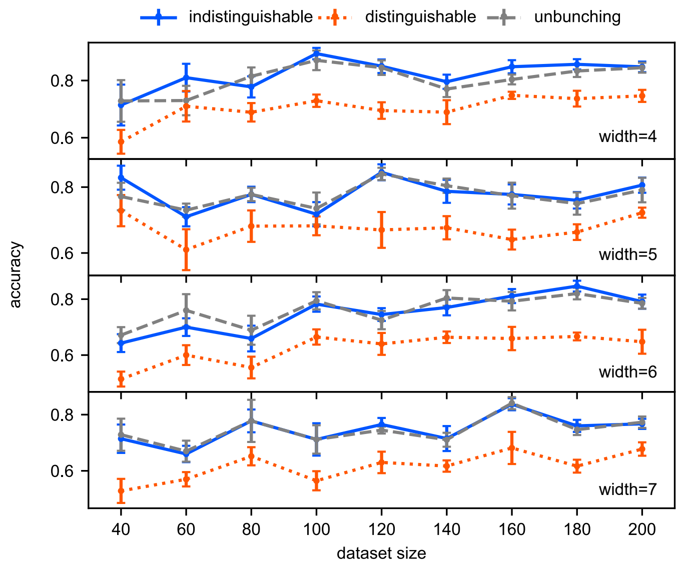

Supplementary Figure S2. **Classification accuracies under approximation using unbunching photons** Simulation of the classification accuracies using quantum kernels, both considering and not considering bunching events and classical kernels, varying different circuit widths. The error bars show the standard deviation of the test accuracies, obtained over 5 repetitions of the simulations with different random seeds.

Supplementary Note 3. APPROXIMATION OF BOSONIC KERNELS WITH POST SELECTION

The inner product in bosonic kernels K_Q and K_C can be measured experimentally by collecting the probability over all output photon states $\{\phi_{m,n}\}$, which corresponds to the normalization of coincidence counting for all possible configuration. Here m is the mode number of the quantum circuit $U(\theta)$ with parameters θ and n is the photon number. For example, for an input state s and a output state t,

$$P_\theta(t|s) = \frac{\mathrm{CC}(t)}{\sum_{k\in\{\phi_{m,n}\}} \mathrm{CC}(k)} \tag{S14}$$

including both unbunching (collision-free) photon states $\mathbb{U}$ and bunching photon states $\mathbb{B}$, $\{\phi_{m,n}\} = \mathbb{U} \oplus \mathbb{B}$, which requires photon number resolving detectors for each output mode. Since we are unable to detect those bunching events, a common solution to this issue is to only keep the statistics of unbunching events [3], when all photons output from the circuits are detected in separate modes. We normalize the probabilities of unbunching states $\mathbb{U} \subsetneq \{\phi_{m,n}\}$:

$$P_\theta(t|s) \approx P_\theta^{\mathbb{U}}(t|s) = \frac{\mathrm{CC}(t)}{\sum_{k\in\mathbb{U}} \mathrm{CC}(k)} \tag{S15}$$

[4, Section 13] As shown in (7) the unbunching probabilities are a good estimate of the ideal bosonic statistics when $m >> n^2$. We can use the these statistics to define the *unbunching kernel*:

$$K(x_i, x_j) \approx K_U(x_i, x_j) = P_\theta^{\mathbb{U}}(\psi|\psi) = \frac{\mathrm{CC}_\psi}{\sum_{1\le i<j\le 6} \mathrm{CC}_{ij}} \tag{S16}$$

for some state $\psi \in \mathbb{U}$.

Under this approximation, the unbunching kernel is not a positive definite kernel since the estimated probability is not real inner products in a Hilbert space. In practice however, the function can still be used in a support vector machine and our simulations show that the resulting leaning algorithm performs similarly to the bosonic kernel, see Fig S2.

In this case, the labeling is still optimized for the quantum kernel K_Q with respect to the classical kernel K_C. We observe that the unbunching kernel performs similarly to the case where all of the output events are considered and consistently better than classical particles. This indicates this approximation is good enough to reproduce the quantum kernel by detecting only the collision-free photons.

Supplementary Note 4. INTERMEDIATE DEGREES OF PHOTON INDISTINGUISHABILITY

To test the role of photon indistinguishability, we change the relative temporal delay between the input photons, making them distinguishable in their arrival times. This implies that, for delays that are lower than the coherence time of photons, we are sending the following mixture in the chip: $r\,|1,1,0,0,0,0\rangle + (1-r)\,|1,1',0,0,0,0\rangle$, with $0 < r < 1$. Here, r is defined as the *degree of indistinguishability* [5]. We report this transition, with the corresponding test accuracies for a data set of 40 elements in Fig. S3. These values are obtained for datasets of 40 elements, where 2/3 are used as training and 1/3 as test set, with input state $|0,0,1,1,0,0\rangle$.

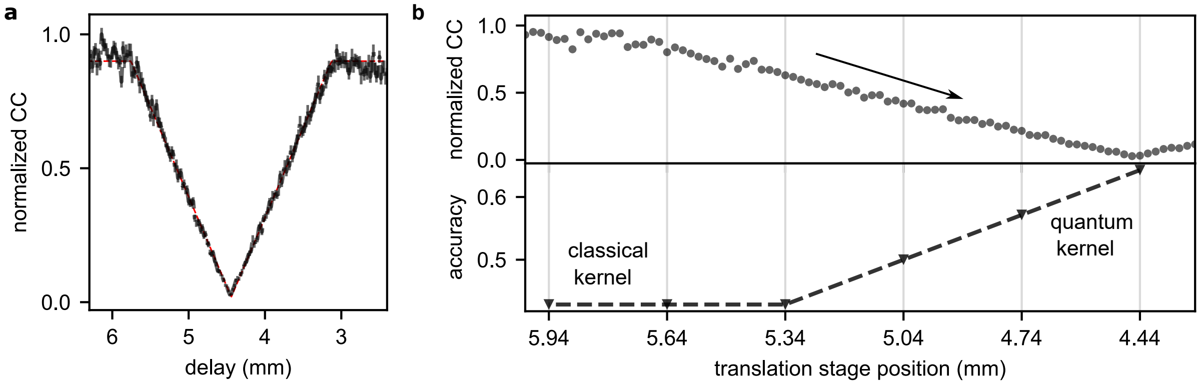

Supplementary Figure S3. **Classification test accuracy with respect to degree of photon indistinguishability. a**. On-chip Hong-Ou-Mandel interference. The maximal visibility achieved amounts to 0.9720±0.0044. The noise in the plot comes from the translation stage and photon number counting in a single experiment. The red line is the obtained fitting triangular function. The measurements were taken with an integration time of 5 s, with a detection rate of 10 kHz. **b**. Transition from fully distinguishable to fully indistinguishable photon inputs.

Supplementary Note 5. NUMERICAL EXPERIMENTS

A. Dataset generation algorithm

In the Algorithm 1, we report the algorithm used for all simulation mentioned in this work.

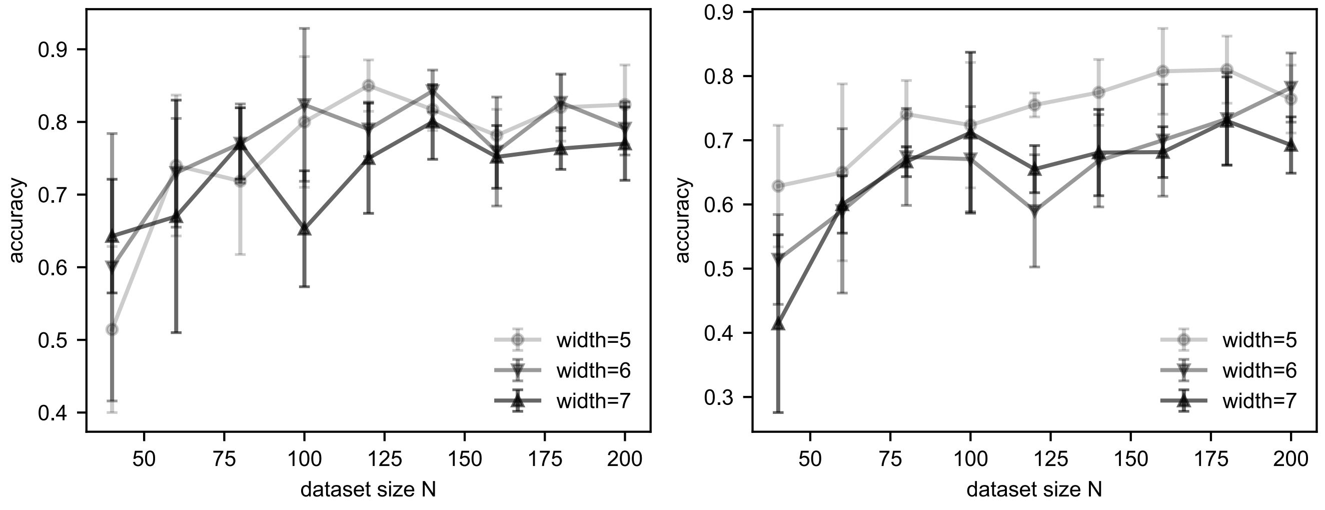

Supplementary Figure S4. **Accuracy of the quantum kernel K_Q against the number of data points for several widths of the integrated circuit.** The two plots refer to two different input states, i.e. on the left, we consider the state $|1,1,0,0,0,0\rangle$ and, on the right, we consider $|0,0,1,1,0,0\rangle$. Each point is the mean accuracy on 5 iterations, each starting with randomly generated data. The standard error on the mean is shown by error bars. For each width, we observe a gradual increase in classification accuracy with number of data points. Typically, lower widths perform better for the same number of data points. We hypothesise that this is because the number of parameters available to the system increases with width, and so too many parameters on too little data leads to data being sparse in the space (i.e. all data points being approximately linearly independent so difficult to classify).

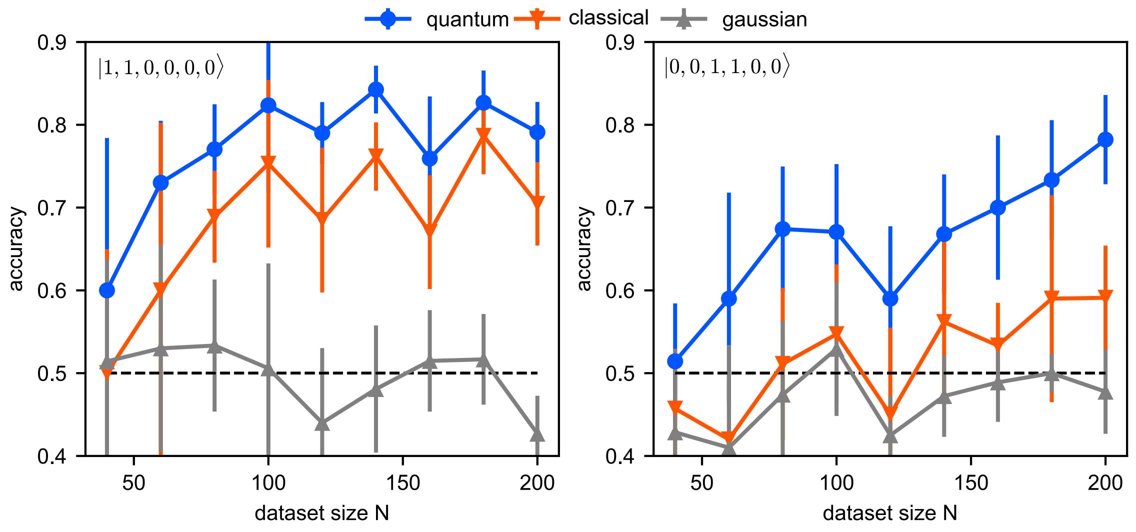

Supplementary Figure S5. **Classification accuracies of photonic and Gaussian kernels.** We show the numerically estimated average test accuracies over 5 iterations for quantum kernel, classical kernel and Gaussian kernels as the number of data points grow. The black dash lines indicate the random guessing accuracy of 50%. The data for the quantum kernel comes directly from Figure S4 – the width that produced the highest accuracy is shown. The standard error on the mean is shown by error bars by five random dataset generation. We observe separation between the classification accuracy of the quantum and classical kernels. This is more prominent for the center initial state (right-hand figure). Both perform considerably better than the Gaussian kernel on the same data, which has an average accuracy of less than 60%.

Algorithm 1 Dataset Generation

input circuit width m, depth k of the chip, number of input photons n, initial state $s \in \Phi_{m,n}$, number of data points N
generate N random data points $\{x_i\}_{i=1}^N, x_i \in \mathbb{R}^d$ ▷ Note d is proportional to mk
$U(x_i) \leftarrow \prod_{j,k\in C} \mathrm{SU}_{\mathrm{MZI}}(x_i^{(j)}, x_i^{(k)})$ ▷ C is the index in Clements coding
▷ $\mathrm{SU}_{\mathrm{MZI}}$ is the matrix of Mach-Zehnder interferometer.
while $0 < i \leq N$ **do** ▷ calculate the Gram matrix
  while $0 < j \leq i$ **do**
    $K_Q(x_i, x_j) \leftarrow |\mathrm{per}(U(x_i, x_j))|^2$
    $K_C(x_i, x_j) \leftarrow \mathrm{per}(|U(x_i, x_j)|^2)$
    $j \leftarrow j + 1$
  end while
  $i \leftarrow i + 1$
end while
$S \leftarrow \sqrt{K_Q}(K_C + \lambda I)^{-1}\sqrt{K_Q}$
$\lambda \leftarrow$ max eigenvalue of S, $\mathbf{v} \leftarrow$ max eigenvector of S
$g_{CQ} \leftarrow \sqrt{\lambda}$
$y \leftarrow \mathrm{sign}(\mathbf{v})$ ▷ apply sign function to get binary labels
output $D = \{(x_i, y_i)\}_{i=1}^N$.

B. Benchmark algorithm

To benchmark the photonic kernels, we choose the following four kernels to compare the classification accuracies, gaussian kernel, polynomial kernel, liner kernel and neural tangent kernel. First, for the gaussian kernel, the Gram matrices are calculated as

$$K_G(x_i, x_j) = \exp\bigl(\gamma |\, x_j - x_i \,|^2\bigr) \tag{S17}$$

Here γ is a hyper parameter and x_i, x_j are two different data points. Second, the polynomial kernel is defined as

$$K_P(x_i, x_j) = (\gamma x_i \cdot x_j + r)^d \tag{S18}$$

where there three hyper-parameters $\gamma r, d$. In both gaussian kernel and polynomial kernel, we run the grid search to find the optimal hyper-parameters. Next, for the linear kernel, we take the simple linear inner product as

$$K_L = x_i \cdot x_j \tag{S19}$$

For the above three kernels, the Gram matrices are processed by a classical support vector machine to predict the data points in the test datasets.

Last but not least, to implement the neural tangent kernel, we adopt the open source proGram [6] which trains an ensemble of infinite width neural networks using gradient descent. The network consists two one input layer, two hidden layers and one output layer. Without loss of generality, the two hidden layers both include 30 neurons, as the input data points are 30 dimensional. The sign function is applied on the output to identify the class. In fact, the hyper parameters in this infinite work does not affect the accuracy as the labeling is very specific to separate the photonic kernels.

C. Simulations

We conduct the following numerical simulations to substantiate the feasibility of our method. The classification accuracy by quantum, classical, gaussian, neural tangent, polynomial and linear kernels are labeled as $a_Q, a_C, a_G, a_N, a_P, a_L$ below.

a. Task1 As a first simulation, we fix photon number n, input state s and dataset size N, and get 3D plots of width m, depth k and accuracy $\{\, a_Q, a_C, a_G \,\}$. Results indicate that width and depth should be linearly related to maximise separation. In subsequent experiments we used $m = k$. We used two types of initial states, Left state $|\psi_L\rangle = |1, 1, 0, 0, 0, 0\rangle$ where photons are input of one side of the chip and Cent state $|\psi_C\rangle = |0, 0, 1, 1, 0, 0\rangle$ where they are input in the middle.

b. Task 2 In our second simulation, we fix photon number n and the encoding state s, set $m = k$ and plot the quantum accuracy a_Q and classical accuracy against the number of data points N. The results are given in Fig. S4. We can observe this point experimentally by checking that $s_Q = g_{CQ}^2 s_C$. When this is violated the Gram matrix K_Q is not invertible, there are too many linear dependencies between the columns, i.e. too many data points in a space of dimension $\binom{n+m-1}{n}$.

c. Task 3 Now we have a way of scaling width m, depth k and number of data points N together, by taking the best chip size $m = k$ for a given number of data points. We plot the accuracies for quantum, classical and gaussian kernels as the system size grows. The results are given in Figure S5.

d. Task 4 We also performed simulations to test unbunching kernels on the datasets resulting from separation between quantum and classical Gram matrices, see Figure S2. Separating directly between unbunching and distinguishable kernels is often not possible. This is because the separation method requires to compute the square root of the Gram matrix, but the one obtained from unbunching statistics is often not completely positive.

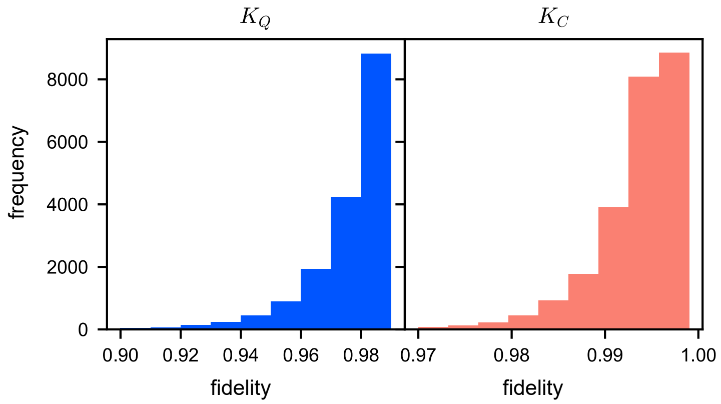

Supplementary Figure S6. **Experimental probability fidelity of the unitaries**. The experimental fidelity for all data points performed by the quantum kernel (blue, left) and the classical kernel (orange, right). .

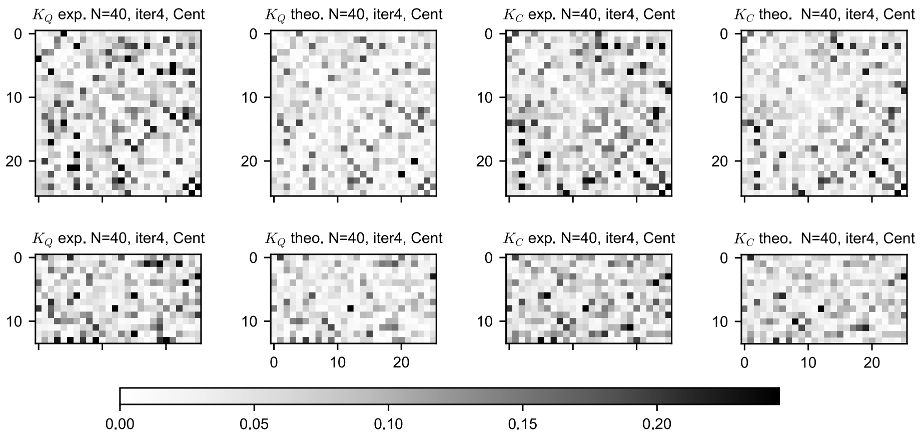

Supplementary Figure S7. **Experimentally estimated Gram matrices** We show the Gram matrices corresponding to the quantum kernel (left) and the ones referring to the classical kernel (right), compared to the theoretical predictions. The number of data points is 40, and the encoding state is $|0, 0, 1, 1, 0, 0\rangle$. The top row shows the inner products of training Gram matrices and the bottom row shows the train-test Gram matrices.

Supplementary Note 6. EXPERIMENT METHODS

As described in the main text, the experimental statistics of was post processed to calculate each element of the Gram matrices. Since the Gram matrices are symmetric, $K_{ij} = K_{ji}$, thus for N data points, we run $N(N-1)/2$ times to get the Gram matrices. In Figure 3**c**, for each dataset size varying from 40 to 100, we have 5 independent

iterations, this indicates 53,300 running of unitary for both quantum and classical kernels. The total experiments lasted hundreds of hours.

The fidelities obtained from the implementation of unitaries are reported in Figure S6. The mean fidelity is 0.9816±0.0148 and 0.9934±0.0048, for the quantum kernel and classical kernel respectively. To illustrate the difference between two kernels, an example of estimated Gram matrices is shown in Figure S7.

To understand the impact of phase noise and imperfections in our experiments, we also performed the following simulations to verify the robustness of fidelity estimated used in the experiment. For all the simulations we run 500 random unitaries to estimate the fidelity, between the original unitary and the one perturbed by the noise, including the phase noise, bias of beam splitter and photon statistics respectively.

1. Phase noise in the MZI. In our setup, we use a 16 bit current output to drive every thermal phase shifter. The current output range is 24 mA and the corresponding current resolution is 0.003 mA. Considering that the average current to drive a 2π phase is around 12 mA, the phase resolution is 0.0015 rad. Moreover, let us note that, in our experiment, we do not use active temperature control and the temperature fluctuation is around 0.1 degree. To account for this kind of noise, we sample random phases from a normal distribution, around the correct value, with a standard deviation of 0.01 0.005 0.001 0.0005 and 0.0001 rad, respectively. The results are shown in Fig. S8a.

2. Bias of beam splitter. The ideal beam splitter, making up each MZI, should be 50:50, but the real devices used in our chip are directional couplers with large curvature. To account for such imperfection, we consider our directional couplers as tunable Mach-Zehnders whose internal phase has an offset and amounts to $\pi/4 + \theta_{\text{bias}}$, rather than $\pi/4$.

$$U_{\text{BS with bias}} = \begin{pmatrix} \cos(\pi/4 + \theta_{\text{bias}}) & -\sin(\pi/4 + \theta_{\text{bias}}) \\ \sin(\pi/4 + \theta_{\text{bias}}) & \cos(\pi/4 + \theta_{\text{bias}}) \end{pmatrix} \tag{S20}$$

   Similarly to before, we select the bias, drawing them from a Gaussian distribution, centered at $\theta = 0$ and with a standard deviation of 0.05, 0.03, 0.02, 0.01, 0.005 rad. The results are shown in Fig. S8b.

3. Finite photon statistics. Considering that the underlying distribution for photon counting is Poissonian, every term in Eq. S14 has standard deviation $\Delta N_{\text{CC}} = \sqrt{N_{\text{CC}}}$. To estimate the impact of such noise, we performed a Monte Carlo simulation, considering different total coincidence countings, i.e. 100, 500, 1000, 5000, 10000, and created random matrices to sample the coincidence counting per channel. The obtained fidelity estimations are shown in Fig. S8c.

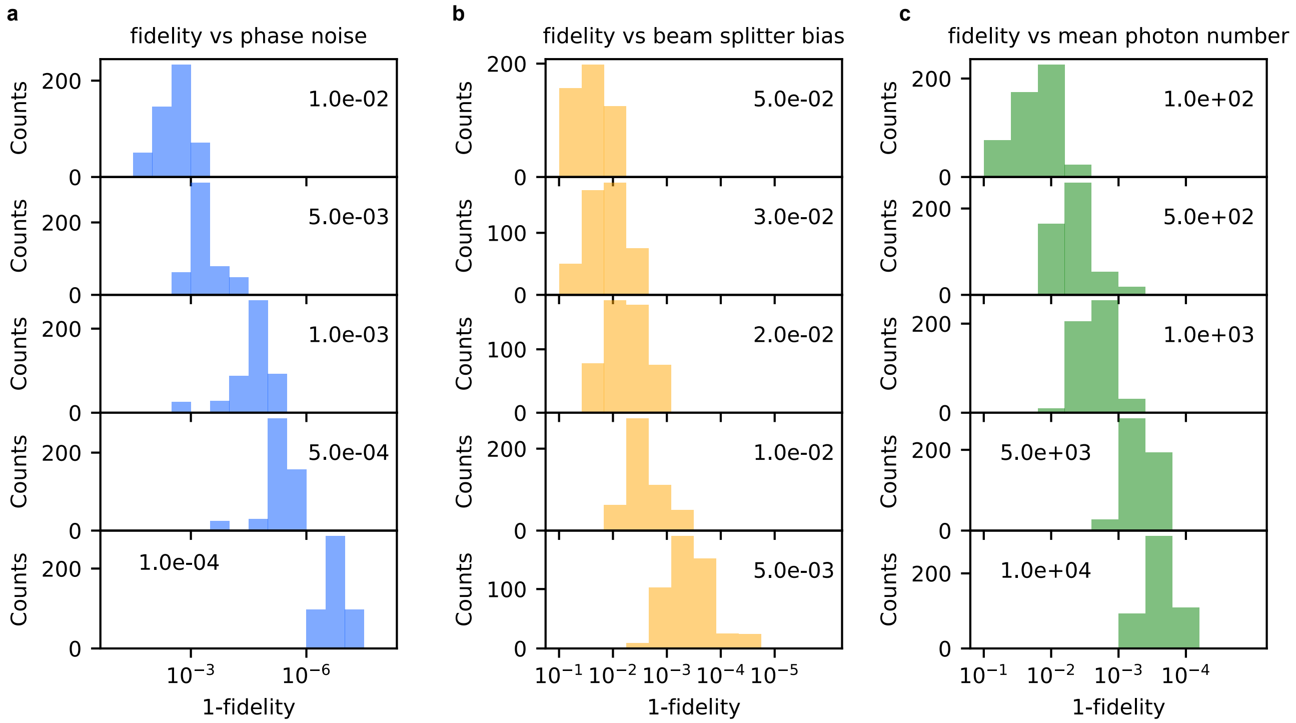

Supplementary Figure S8. **Robustness of fidelity estimation**. The fidelity estimation of the implemented unitaries under three different noise types: phase noise in the MZI shown in **a**, beam splitter bias shown in **b**, and photon statistics shown in **c**. Each graph plots the histogram of fidelity between containing 500 random unitaries with noise (obtained sampling from a Gaussian distribution centered at the ideal value and with the standard deviation written in the plots) and without noise.

SUPPLEMENTARY REFERENCES

[1] Banchi, L., Kolthammer, W. S. & Kim, M. Multiphoton Tomography with Linear Optics and Photon Counting. *Physical Review Letters* **121**, 250402 (2018). URL `https://link.aps.org/doi/10.1103/PhysRevLett.121.250402`.

[2] Huang, H. Y. *et al.* Power of data in quantum machine learning. *Nature Communications* **12** (2021). URL `http://dx.doi.org/10.1038/s41467-021-22539-9`. ArXiv: 2011.01938 Publisher: Springer US.

[3] Tillmann, M. *et al.* Experimental boson sampling. *Nature Photonics* **7**, 540–544 (2013). ArXiv: 1212.2240.

[4] Aaronson, S. & Arkhipov, A. The Computational Complexity of Linear Optics. *Proceedings of the 43rd annual ACM symposium on Theory of computing - STOC '11* **9**, 333 (2010). URL `http://portal.acm.org/citation.cfm?doid=1993636.1993682`. ArXiv: 1011.3245 Publisher: ACM Press Place: New York, New York, USA ISBN: 9781450306911.

[5] Renema, J. J. *et al.* Efficient Classical Algorithm for Boson Sampling with Partially Distinguishable Photons. *Physical Review Letters* **120**, 220502 (2018). URL `https://doi.org/10.1103/PhysRevLett.120.220502`. Publisher: American Physical Society.

[6] Novak, R. *et al.* Neural tangents: Fast and easy infinite neural networks in python. In *International Conference on Learning Representations* (2020). URL `https://github.com/google/neural-tangents`.
